# Insights into the Molecular Mechanisms of Protein-Ligand Interactions by Molecular Docking and Molecular Dynamics Simulation: A Case of Oligopeptide Binding Protein

**DOI:** 10.1155/2018/3502514

**Published:** 2018-12-04

**Authors:** Yi Fu, Ji Zhao, Zhiguo Chen

**Affiliations:** ^1^Wuxi Research Center of Environmental Science and Engineering, Wuxi 214153, Jiangsu, China; ^2^School of Internet of Things Engineering, Wuxi City College of Vocational Technology, Wuxi 214153, Jiangsu, China; ^3^School of Internet of Things Engineering, Jiangnan University, Wuxi 214122, Jiangsu, China

## Abstract

Protein-ligand interactions are a necessary prerequisite for signal transduction, immunoreaction, and gene regulation. Protein-ligand interaction studies are important for understanding the mechanisms of biological regulation, and they provide a theoretical basis for the design and discovery of new drug targets. In this study, we analyzed the molecular interactions of protein-ligand which was docked by AutoDock 4.2 software. In AutoDock 4.2 software, we used a new search algorithm, hybrid algorithm of random drift particle swarm optimization and local search (LRDPSO), and the classical Lamarckian genetic algorithm (LGA) as energy optimization algorithms. The best conformations of each docking algorithm were subjected to molecular dynamic (MD) simulations to further analyze the molecular mechanisms of protein-ligand interactions. Here, we analyze the binding energy between protein receptors and ligands, the interactions of salt bridges and hydrogen bonds in the docking region, and the structural changes during complex unfolding. Our comparison of these complexes highlights differences in the protein-ligand interactions between the two docking methods. It also shows that salt bridge and hydrogen bond interactions play a crucial role in protein-ligand stability. The present work focuses on extracting the deterministic characteristics of docking interactions from their dynamic properties, which is important for understanding biological functions and determining which amino acid residues are crucial to docking interactions.

## 1. Introduction

Molecular docking methods are of utmost importance and have been widely used in new drug design and discovery projects [1–3]. Molecular docking methods can provide a relatively fast and economical alternative to standard experimental techniques [4, 5]. They aim to predict the experimental binding modes and affinities of small molecules within the binding site of particular receptor targets. Two important goals in molecular docking are to find correct binding poses and to accurately predict binding affinity. More accurate predictions of binding poses and binding affinities can suggest candidates for active compounds with higher true positive rates and can considerably reduce expensive experimental efforts [6]. The quality of molecular docking depends on two factors: the optimization search method and the scoring function [7–9]. An optimization algorithm mainly detects docking conformations with minimum binding energies. The scoring function is used to evaluate the results obtained from the search. In this article, we used AutoDock 4.2 software to perform molecular docking. In AutoDock 4.2, a new hybrid algorithm of random drift particle swarm optimization with local search (LRDPSO) [10] and Lamarckian genetic algorithm (LGA) [11] were used as energy search algorithms. Here, random drift particle swarm optimization (RDPSO) algorithm is a variant of the particle swarm optimization algorithm (PSO). LGA is a combination of a genetic algorithm (GA) and a local search (LS) and is one of the classical energy search algorithms in AutoDock 4.2 software.

In this study, oligopeptide binding protein (OppA) [12] was used as the experimental object. OppA can act as a receptor for peptide transport across the cell membrane and is a potential target in antibacterial drug design [13]. It has broad specificity and can bind to a wide range of peptides that are 2–5 amino acid residues long. Here, we used the sequence Lys-Tyr-Lys as the ligand binding structure. The OppA structure (PDB code 1B58 [14]) consists of three domains: domain I contains residues 1–44, 189–269, and 487–517; domain II contains residues 45–188; and domain III contains residues 270–486. Domains I and II have a bilobate structure, allowing the ligand to act as a flexible hinge connecting the two lobes. In the protein-ligand complex structure, ligands are completely contained in the protein interior [15]. For LRDPSO and LGA, we selected the two best solutions for 1B58 in terms of binding energy. The lowest binding energies in LRDPSO and LGA were −25.09 kcal/mol and −12.74 kcal/mol, respectively.

Protein-ligand interactions play an important role in most biological processes, such as signal transduction, cell regulation, and immune response [16, 17]. Studying protein-ligand interactions continues to be very important in life science fields [18–21]. There are variations in protein-ligand complex structures due to different docking methods. In this article, we mainly focus on analyzing the binding interactions between a protein and a ligand, especially on the divergence of protein-ligand interactions, which can help us understand and address key questions, such as those related to the diversity of binding affinity and specificity. We have performed molecular dynamic (MD) simulations on molecular docking results at four different temperatures (ranging from 300 K to 600 K) to establish a more reliable mechanism for illustrating ligand-protein interactions. The dynamic properties of complexes have been compared in terms of residue flexibility, binding energy, salt bridge, and hydrogen bond interactions in the binding region and structural variations during unfolding at different temperatures. This study provides a better understanding of the specific interactions predicted by different docking methods, and it also allows us to more precisely study a binding site or region to increase docking accuracy.

## 2. Materials and Methods

### 2.1. Protein and Ligand Structure Preparation

The OppA structure (PDB code 1B58 [14]), which was obtained from the RSCB protein data bank (http://www.rcsb.org/), was used as a receptor of the experimental object. The sequence Lys-Tyr-Lys, which contains 43 atoms, was used as the ligand structure. The docking results were used as models for the MD simulation.

### 2.2. Genetic Algorithm (GA)

AutoDock has been applied with great success in the prediction of binding conformations of protein-protein interactions, peptide-antibody complexes, and enzyme-inhibitor complexes. Earlier versions of AutoDock used simulated annealing as a search method; a subsequent version added the options of a GA, a LS method, and a combination of GA and LS, which is called LGA. In LGA, a GA is used for global searching, and Solis and Wets [22] is used as the LS method. Each generation is followed by a LS, which is performed on a user-defined proportion of the population.

Genetic algorithm [23, 24] is a population-based metaheuristic algorithm, which contains initial population generation, fitness function evaluation, iteration, and termination condition check off four steps. In addition, every iteration step includes selection, crossover, and mutation operations. The pseudocode of GA is described in Algorithm 1.

### 2.3. Hybrid Algorithm of RDPSO and Local Search (LRDPSO)

RDPSO [25] is derived from canonical PSO trajectory analysis [26] and the free electron model. Particle behavior in RDPSO is assumed to be similar to an electron moving in a metal conductor in an external electric field. Particle movement is thus the superposition of thermal and drift motions, which is based on a global search and local search of the particle, respectively.

In a RDPSO with *M* individuals, each individual is treated as a volumeless particle in the *N*-dimensional space. *V*_*i*,*n*_=(*V*_*i*,*n*_^1^, *V*_*i*,*n*_^2^,…, *V*_*i*,*n*_^*N*^) and *X*_*i*,*n*_=(*X*_*i*,*n*_^1^, *X*_*i*,*n*_^2^,…, *X*_*i*,*n*_^*N*^) are expressed as the velocity vector and the position vector of particle *i* at the *n*^th^ iteration, respectively.

According to the above model, the updated equations of RDPSO can be expressed by the following equation:

(1) $$\begin{array}{c} V_{i,n+1}^{j}=\alpha \left|C_{n}^{j}-X_{i,n}^{j}\right|\phi_{i,n}^{j}+\beta \left(p_{i,n}^{j}-X_{i,n}^{j}\right), \end{array}$$

(2) $$\begin{array}{c} X_{i,n+1}^{j}=X_{i,n}^{j}+V_{i,n+1}^{j}, \end{array}$$

(3) $$\begin{array}{c} C_{n}^{j}=\left(\frac{1}{M}\right)\overset{M}{\underset{i=1}{\sum }}P_{i,n}^{j}, \end{array}$$

for *i*=1,2,…, *M*; *j*=1,2,…, *N*, where *α* > 0 is a parameter called the thermal coefficient and *β* > 0 is another parameter called the drift coefficient. *ϕ*_*i*,*n*_^*j*^ is a random number, *ϕ*_*i*,*n*_^*j*^ ~ *N*(0,1). *p*_*i*,*n*_^*j*^ is the local attractor of PSO algorithm. *P*_*i*,*n*_^*j*^ is the personal best (*pbest*) position of particle *i*. *C*_*n*_^*j*^ is defined by the mean of the *pbest* positions of all particles, called the mean best (*mbest*) position. The pseudocode of RDPSO is described in Algorithm 2.

The hybrid of the RDPSO algorithm with the Solis and Wets algorithm together form the LRDPSO algorithm. Here, the RDPSO algorithm is used as a global search algorithm, and the Solis and Wets algorithm is used as a local search algorithm. The Solis and Wets algorithm can facilitate torsional space search, since it does not require gradient information about the local energy landscape [22, 27]. The addition of local search effectively maintains the diversity of particles and prevents premature convergence of the algorithm. Therefore, the effective combination of the RDPSO algorithm and the Solis and Wets algorithm can provide a good global search ability and a rapid convergence ability. The pseudocode of LRDPSO is described in Algorithm 3.

### 2.4. The Docking Experiment Settings

In molecular docking experiments, the prepared protein and ligand structures were saved in the PDBQT file format. The AutoDockTools (ADT) was used as molecular graphical visualization tool. The AutoDock package includes AutoGrid program and AutoDock program. AutoGrid program is responsible for the calculation of energy grid maps; here, a grid size was set to 60 × 60 × 60 points with a spacing of 0.375 Å. AutoDock program is responsible for the conformation search and energy evaluation; here, for LRDPSO and LGA algorithms, the initial population was set to 50 individuals, the number of energy function evaluations was set to 2.5 × 10^5^, and maximum number of generations was set to 27,000. The detail setting information refers to reference [10]. We followed the methods of Fu et al. [10]. In that article, we concentrate on discussing the design of LRDPSO algorithm and its application in protein-ligand docking.

### 2.5. Molecular Dynamic (MD) Simulation

Docked protein-ligand complexes were subjected to molecular dynamic simulations using NAMD software [28]. MD simulations were performed using the CHARMM27 force field [29]. Visual molecular dynamics (VMD) [30] was used to generate PSF files for both complexes. Both complexes were solvated in cubic water boxes containing transferable intermolecular potential with 3 points (TIP3P) water molecules [31]. The box size was chosen so that there was a distance of 10 Å between the protein surface and the edges of the periodic box. A 12 Å cutoff distance was used to calculate short-range nonbonded interactions. The particle mesh Ewald (PME) [32] method was used to calculate long-range electrostatic interactions. The SHAKE method [33] was used to constrain all bonds involving hydrogen atoms. The system first performed 10000 steps of steepest descent with energy minimization. Then, the minimized system was used to perform simulations using an NVT ensemble. The Nosé–Hoover method [34] was used to maintain a constant temperature. The simulated temperature was set in the range of 300 K to 600 K with an interval of 100 K. The simulation time for each simulated temperature was set to 10 ns. The time step of each simulation was set to 2 fs. Visualizations and data analysis were performed with VMD software.

## 3. Results and Discussion

### 3.1. Molecular Docking Energy Analysis

A semiempirical free energy scoring function was used to evaluate a docked conformation in AutoDock 4 [11]. The total docked energy of the ligand and protein included two components, which are the intramolecular energy and the intermolecular energy. The intramolecular energy was evaluated for the transition from the unbound state to the bound conformation of the ligand and protein. The intermolecular energy was estimated for the combination of the ligand and the protein in their bound conformation.

The force field consists of six pairwise evaluations (*V*) and an estimate of the conformational entropy lost upon binding (Δ*S*_conf_):

(4) $$\begin{array}{c} \Delta G=\left(V_{\text{bound}}^{L-L}-V_{\text{unbound}}^{L-L}\right)+\left(V_{\text{bound}}^{P-P}-V_{\text{unbound}}^{P-P}\right)+\left(V_{\text{bound}}^{P-L}-V_{\text{unbound}}^{P-L}+\Delta S_{\text{conf}}\right), \end{array}$$

where *P* refers to the “protein” and *L* refers to the “ligand” in a protein-ligand docking calculation.

Each of the pairwise energetic items includes the following energy: the first item is a Lennard-Jones 12-6 van der Waals interaction, the second item is a 12-10 hydrogen bond potential, the third item is a coulombic electrostatic potential, and the final item is a desolvation potential.

(5) $$\begin{array}{c} V=W_{\text{vdw}}\underset{i,j}{\sum }\left(\frac{A_{ij}}{r_{ij}^{12}}-\frac{B_{ij}}{r_{ij}^{6}}\right)+W_{\text{Hbond}}\underset{i,j}{\sum }E\left(t\right)\left(\frac{C_{ij}}{r_{ij}^{12}}-\frac{D_{ij}}{r_{ij}^{10}}\right)+W_{\text{elec}}\underset{i,j}{\sum }\frac{q_{i}q_{j}}{\varepsilon \left(r_{ij}\right)r_{ij}}+W_{\text{sol}}\underset{i,j}{\sum }\left(S_{i}V_{j}+S_{j}V_{i}\right)e^{\left(-r_{ij}^{2}/2\sigma^{2}\right)}. \end{array}$$

**Figure 1:**
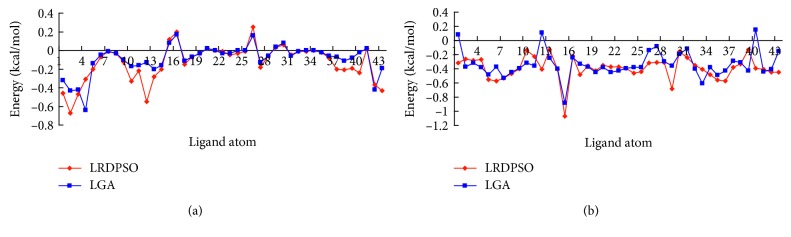
Comparison of the binding electrostatic (a) and van der Waals (b) energy of the ligand atom corresponding to LRDPSO and LGA.

**Figure 2:**
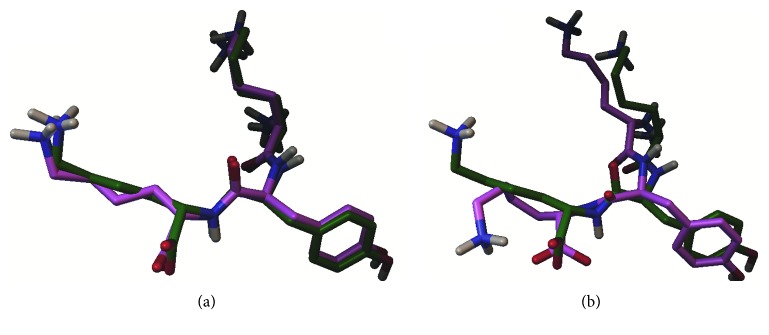
Molecular docking results: superposition of the predicted conformation (shown in magenta) and the native conformation (shown in green). (a) LRDPSO docking method and (b) LGA docking method.

We used the sequence Lys-Tyr-Lys, which contains 43 atoms, as the ligand structure.  Table 1 shows a comparison of the best solutions obtained (out of 30 independent runs) from both LRDPSO and LGA for the OppA complex.  Figure 1 shows a comparison of each ligand atom binding electrostatic energy and van der Waals energy. In general, as indicated in Table 1 and Figure 1, the electrostatic and van der Waals energy of the complex were lower when docked by LRDPSO than when docked by LGA. In the molecular docking predicted complex, a lower binding energy was assumed to be closer to the native state of the complex. For both complexes, the LRDPSO complex was energetically more stable than the LGA one given the obtained energy results; the lowest binding energy corresponded to −25.09 kcal/mol for the LRDPSO complex structure and −12.74 kcal/mol for the LGA one. Both ligands were docked to the site of protein 1B58, but the LRDPSO ligand conformation had a better docking position and a root mean square deviation (RMSD) of 0.63 Å. The RMSD of the LGA docking was 2.00 Å (Figure 2).

For LRDPSO and LGA, we selected the two best solutions with the lowest binding energy in analysis. The ligand conformations in LRDPSO and LGA and the reference ligand are compared in Figure 2.  Figure 2(a) shows the best energy solution (the ligand in magenta) obtained by LRDPSO for protein 1B58 and the reference ligand (in green).  Figure 2(b) shows the solution selected from LGA and the reference ligand. As shown, the ligand has a better conformation in Figure 2(a) than in Figure 2(b) given that the ligand conformation obtained by LRDPSO is very similar to that of the reference ligand. The RMSD scores of the ligand conformations by LRDPSO and LGA were 0.63 Å and 2.00 Å, respectively.

### 3.2. Structural Stability Analysis upon Ligand Binding

We assessed the residue RMSD to study the residue behavior of the protein during the simulations. In general, a residue's RMSD value was considered to represent the local flexibility of a protein. It reflected the mobility of an atom during the MD simulation trajectory. Therefore, a higher residue RMSD value indicated higher mobility; conversely, a lower residue RMSD value indicates lower mobility.

**Figure 3:**
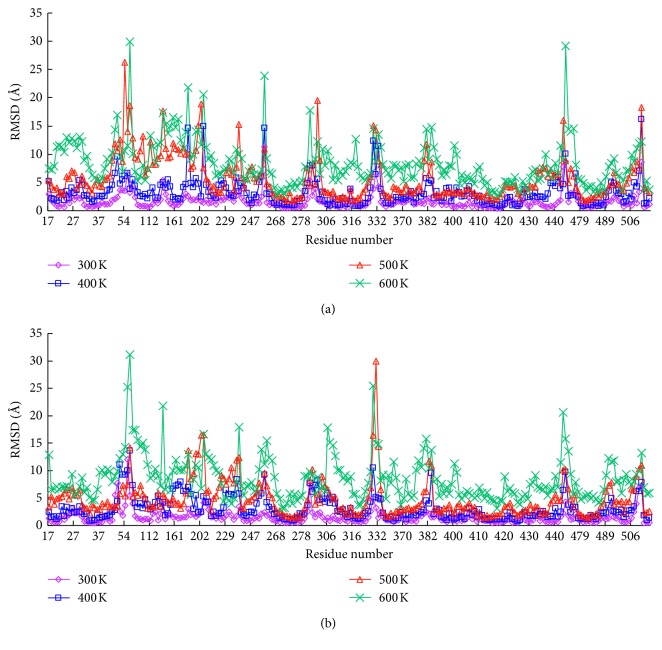
Plot of RMSD and the residue number of the docked complex in the MD simulated structures at different temperatures. (a) LRDPSO-docked complex and (b) LGA-docked complex.

The values of RMSD against each residue were calculated for both complexes by MD simulation at multiple temperatures. This presentation clearly highlighted the differences in some residues of the complexes. The results are shown in Figure 3; for both complexes, there were relatively small bump-like peaks for the structures at 300 K. As the temperature increased, some regions showed significant increases in fluctuation. The curves observed for both complexes exhibited great similarity in their fluctuation trends. Only a few residues showed a great difference in heat fluctuations. For example, when the simulation temperature was 300 K, the RMSD values of residue PHE253 were 11.31 Å and 2.31 Å, corresponding to the LRDPSO and LGA docking results. The RMSD values of residue PHE44 in the LRDPSO and LGA docking complexes were 2.30 Å and 7.65 Å, respectively. In the 500 K simulation, the region between ARG41 and VAL164 had higher fluctuation in the LRDPSO docking complex than in the LGA docking result. In addition, for both complexes, the RMSD values of the residues associated with the ligand were relatively low, even in the high temperature simulations.

### 3.3. Salt Bridge Analysis of the Binding Domain

We docked the tripeptide into the OppA crystal structure. The peptide was bound to the central cleft that surrounded domain I and II. In the case of the OppA complex, there was a difference between the results obtained by LRDPSO and LGA. The difference can be explained by the use of different stochastic search algorithms. Next, we analyze concrete binding interactions and the changes in these interactions under thermal stress.

**Figure 4:**
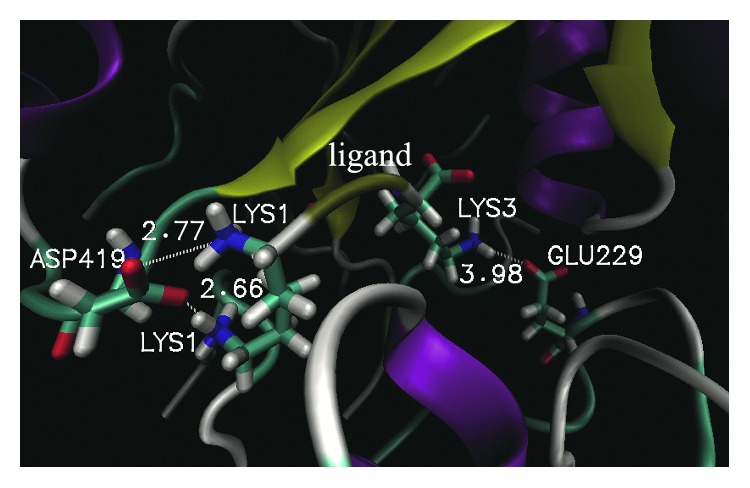
An overview of the location of salt bridges in the binding domain of the LRDPSO-docked complex. The dashed line represents a salt bridge interaction. The adjacent number is the corresponding distance of the salt bridge.

In this article, a salt bridge was defined according to the criterion that the distances between any of the nitrogen atoms of basic residues and the oxygen atoms of acidic residues were less than 4 Å.  Figure 4 shows a salt bridge interaction associated with the ligand and the surrounding protein residues. The ligand can pack tightly into the binding site through a number of favorable salt bridge interactions with the protein. In the LRDPSO docking complex, the LYS1 of the ligand is anchored through a salt bridge interaction with ASP419. Meanwhile, the ligand LYS3 also formed a salt bridge interaction with GLU229 in the complex. Two significant salt bridges (ASP419-LYS1 and GLU229-LYS3) were found in the binding position docked by LRDPSO. Compared to the complex that was docked by LRDPSO, there was only one salt bridge (ASP419-LYS1) located in the binding region in the LGA-docked complex.

**Figure 5:**
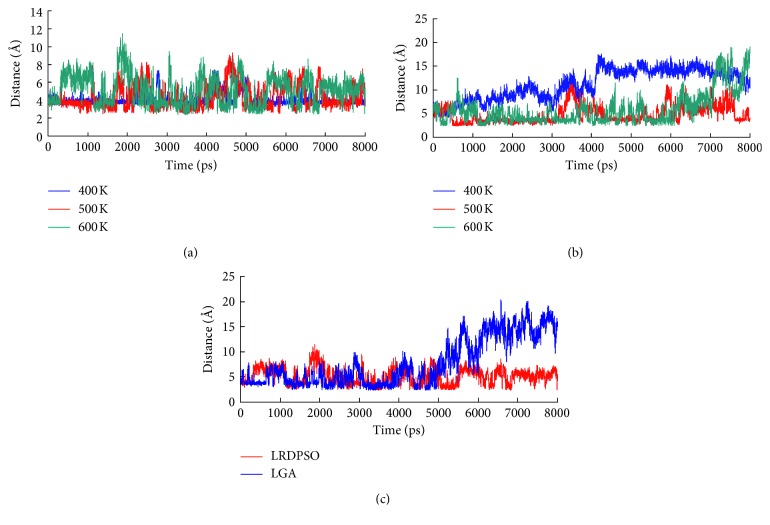
Plot of salt bridge changes in the binding domain as a function of time. (a) Salt bridge ASP419-LYS1 changes in the LRDPSO-docked complex during the simulation from 400 K to 600 K; (b) salt bridge GLU229-LYS3 changes in the LRDPSO-docked complex during the simulation from 400 K to 600 K; (c) comparison plot of salt bridge ASP419-LYS1 in both complexes during the 600 K simulation.

Figure 5 shows the changes in the salt bridge distance in the last 8 ns of the simulations. In the dynamic simulation at 300 K, salt bridge ASP419-LYS1 was stable during the simulation in both complexes. In the 400 K simulation, the salt bridges of both complexes experienced a short separation during the simulation, but they were mostly maintained at a distance of approximately 4.0 Å. In comparison, salt bridge ASP419-LYS1 was found to be more stable in LRDPSO docking than in LGA docking. The average distance of ASP419-LYS1 in the LRDPSO and LGA docking was 3.98 Å and 4.18 Å, respectively. Along with the increase in the simulated temperature, the distance of the salt bridge also changed. In the 500 K simulation, ruptures and restorations of salt bridge ASP419-LYS1 were observed along the whole simulation process. The most obvious difference in this salt bridge between the two complexes was that salt bridge ASP419-LYS1 in the LGA docking was completely separated during the last 5 ns of the simulation. The disruption of salt bridge ASP419-LYS1 probably greatly weakened ligand binding under extremely high temperatures.

The other salt bridge (GLU229-LYS3) was found in only the LRDPSO docking result. Among the four different temperatures, the salt bridge was the most unstable in the 400 K simulation, and it was maintained within a short distance during only the first 400 ps of the simulation. Although the salt bridge plot showed several transient separations in the 500 K and 600 K simulations, the salt bridge remained at a short distance most of the time. Finally, the salt bridge was completely separated after 6.5 ns of simulation at 600 K.

### 3.4. Hydrogen Bond Analysis upon Ligand Binding

Hydrogen bonds are another important factor that influences protein stability. Here, a distance cutoff of 3.5 Å and an angle cutoff of 30° were applied in the hydrogen bond calculation. The study showed that the ligand was entirely buried in the interior of OppA. The main chain and side chain of the ligand could form strong hydrogen bond interactions with the binding site residues of OppA.

**Figure 6:**
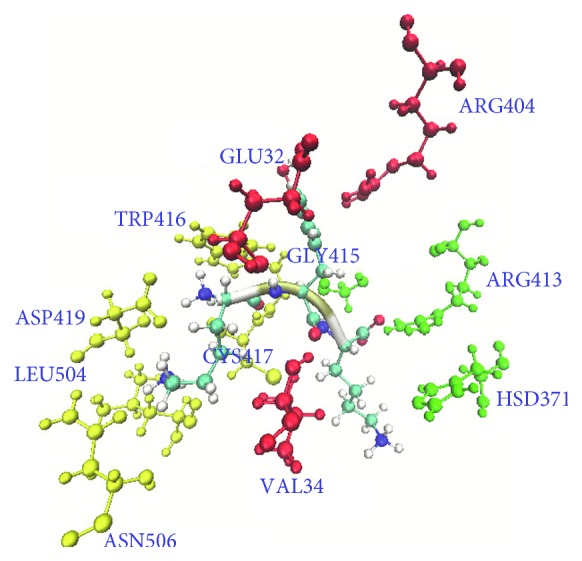
Critical residues of the hydrogen bonds in the binding domain of the LRDPSO-docked result. Residues are shown as ball and stick models, and the ligand is shown in new cartoon secondary structure style. The residues are shown in different colors: yellow for the hydrogen bond network of LYS1 of the ligand, red for the hydrogen bond network of TYR2 of the ligand, and green for the hydrogen bond network of LYS3 of the ligand.

Figure 6 shows the hydrogen bond interactions associated with the ligand and the surrounding protein residues in the LRDPSO docking result. LYS1 forms hydrogen bonds with the side chain of ASP419 and with the main chain of CYS417, and LYS3 forms a hydrogen bond with the side chain of ARG413. In both docking complexes, hydrogen bonds are listed in Tables 2 and 3. Both tables also list the occupancy time of hydrogen bonds in the binding region during the simulation from temperatures of 300 K to 600 K. As shown in the tables, hydrogen bonds are a significant factor that contributes to the stability of protein-ligand binding interactions in both docking complexes. Meanwhile, several hydrogen bond networks exist in the binding region. It can be seen that some hydrogen bonds (listed in the tables) did not exist individually. In the LRDPSO docking result, LYS1 of the ligand could form a hydrogen bond with the receptor residues ASP419, TRP416, CYS417, LEU504, and ASN506, and TYR2 could form a hydrogen bond with GLU32, VAL34, and ARG404. LYS3 formed a hydrogen bond with ARG413, HSD371, and GLY415. These hydrogen bond networks played a positive role in strengthening the binding effect between the protein and ligand.

**Figure 7:**
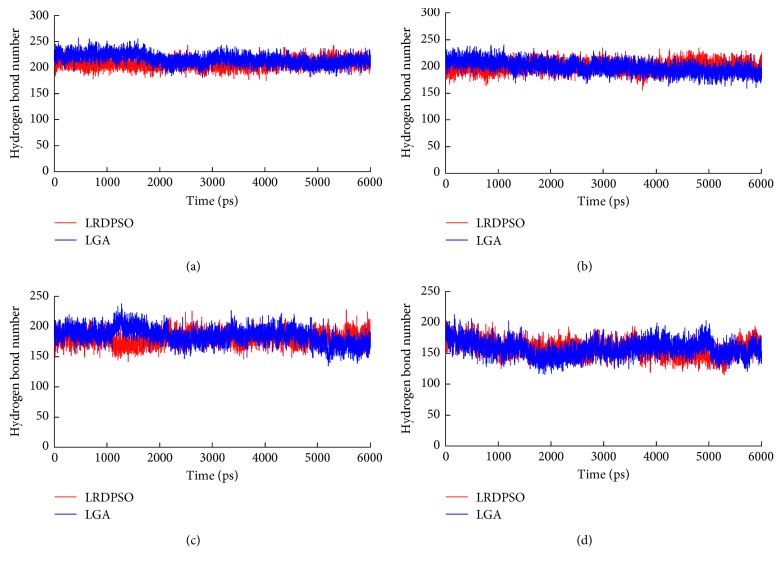
Changes in the hydrogen bond number with respect to simulation time at four different temperatures. Red represents the LRDPSO-docked complex and blue represents the LGA-docked complex. (a) 300 K. (b) 400 K. (c) 500 K. (d) 600 K.

The average number of hydrogen bonds in both complexes is listed in Table 4. As the simulation temperature increased, the protein fold structure was generally weakened, and the structure also became more distorted. These effects resulted in a concomitant decrease in the number of hydrogen bonds at high temperatures.  Figure 7 indicates the number of hydrogen bond changes over simulation time. The number of hydrogen bonds was maintained to a certain extent during the simulations. An interesting finding is that the number of hydrogen bonds did not decrease dramatically with increases in the simulated temperature. When the temperature increased to 600 K, the loss of hydrogen bonds was comparatively higher than at other temperatures. This finding also indicates that the disruption of tertiary structural folds is extremely prominent at a temperature of 600 K.

At the same time, the occupancy time of hydrogen bonds also decreased as the simulation temperature increased. The occupancy time of hydrogen bonds in both complexes is listed over the simulation temperature range of 300 K to 600 K in Tables 2 and 3. The types of hydrogen bonds in the two complexes are almost the same. Only a few hydrogen bonds are different. For the hydrogen bonds that were formed from the same two amino acids, the occupancy time varied in different complexes. For the LRDPSO docking complex, four hydrogen bonds had a high occupancy time (>50%) in the 600 K simulation. However, only two hydrogen bonds in the LGA docking complex had a high occupancy time.

### 3.5. Structural Variation in Unfolding upon Ligand Binding

**Figure 8:**
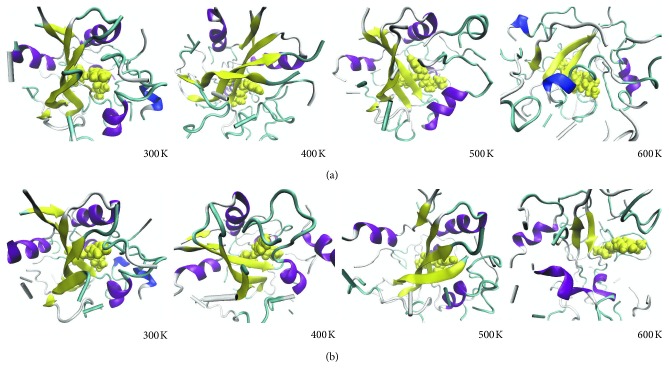
Snapshots from the thermal unfolding simulations for both complexes: (a) LRDPSO-docked complex; (b) LGA-docked complex. The receptor protein structure is represented in a new cartoon secondary structure style, and the ligand is represented in a Vdw style.

Under thermal stress, protein local conformations usually undergo changes. Due to the loss of interactions between residues, a regular secondary structure is often transformed into an irregular secondary structure. The process of unfolding under thermal pressure was not the same in the two complexes as these structures were obtained by different docking methods. There are differences in structural variation during unfolding. Here, an analysis of the time evolution of the secondary structure (Figure 8) can present further structural variation information.

Simulation reveals that the structures of both complexes are very stable when the simulation temperature is 300 K. In the case of the 400 K simulation, only slight structural differences were observed for both complexes. There was high similarity in the structures of the two complexes corresponding to the simulation results at 300 K and 400 K. For the LGA docking result, the complex contained five *α*-helixes at residues VAL34-ASP42, PRO108-TYR115, ASP369-ILE376, TRP397-GLN406, and PRO423-ASN428, and it also contained five *β*-sheets at residues PRO268-ILE277, LEU363-TYR365, ASN394-GLU396, VAL411-CYS417, and ILE479-VAL486. Compared to the LGA docking complex, the LRDPSO docking complex contained four *α*-helixes at residues VAL34-ASP42, TYP112-GLN114, ASP369-ALA375, and TRP397-GLN406, and it also contained six *β*-sheets at residues PRO268-ILE277, LEU312-PRO313, LEU363-ASN366, GLU393-GLN395, VAL411-CYS417, and ALA478-VAL486. In the LRDPSO docking complex, the structure of residues PRO423-ASN428 was switched from an *α*-helix to a loop structure relative to the LGA docking complex.

The structural fluctuations of both complexes were significantly more pronounced in the 500 K simulation. At the end of the simulation, the LGA-docked complex contained four *α*-helixes and four *β*-sheets, whereas the LRDPSO-docked complex contained four *α*-helixes (the same as those in the LGA docking complex) and six *β*-sheets. Compared to the structures before the simulations, some of the *α*-helixes and *β*-sheets were shortened among the regular secondary structures in the high temperature simulations. For regular secondary structures (*α*-helix and *β*-sheet), unfolding begins at the edges and associated turns because the center of these structures is mostly stronger than their edges. In addition, the loops and turns begin to unfold to some extent. Despite these changes, it appears that most native secondary structure elements remained present until the end of the simulation. As shown in Figure 8, the structure of LGA-docked complex was looser than that of LRDPSO-docked complex due to unfolding at 500 K simulation.

At the higher temperature (600 K) simulation, the dominant structural change was that regular structures quickly became disordered, which was accompanied by a loss of molecular contacts. In the LRDPSO docking complex, three *β*-sheets (LEU270-GLU276, ALA412-CYS417, and TYR484-VAL486) and three helixes (ALA110-GLY116, ALA375-ALA377, and PRO423-SER425) maintained stability until the end of simulation. In the LGA docking complex, two *β*-sheets (TYR274-GLU276 and ALA412-ALA414) and four helixes (SER107-TYR109, LEU370-ILE376, PRO423-ASN428, and ASN437-LYS442) were present. Among these helixes, ASN437-LYS442 was reformed by a loop after unfolding of the structure. In other words, the unfolding process generated new 3–10 helixes and *α*-helixes, which originated from areas that were initially coils and loops. The most important point is that the ligand is completely out of the binding position due to the unbinding of ligand and protein in LGA-docked complex.

## 4. Conclusion

In this article, molecular docking and molecular dynamic simulations were performed to provide insights into the structural and dynamic characteristics of OppA-peptide binding interactions. The analysis results reflected the reaction differences between proteins and ligands in the two docking methods. The results showed that although the types of hydrogen bonds in the two complexes were nearly the same, the occupancy time of the same hydrogen bonds was different in the different complexes. For salt bridges, there were two significant salt bridges in the LRDPSO docking result, which were ASP419-LYS1 and GLU229-LYS3. In the LGA-docked structure, there was only one stable salt bridge ASP419-LYS1 located in the binding region. Based on these findings, the electrostatic and van der Waals energy were lower for the ligand docked by LRDPSO than for the ligand docked by LGA. For structural variation under thermal stress, the complex docked by LRDPSO was more stable than the complex docked by LGA at high temperatures. This study provided a concrete difference in OppA-peptide binding based on the two docking methods. It revealed that nonbonded interactions are a significant driving force in biomolecular interactions and stability. It also showed that the contribution of electrostatic interactions is an important factor in binding affinity differences. This study provides a useful guide for drug design and protein engineering and future design studies with the OppA system.

## Figures and Tables

**Algorithm 1:**
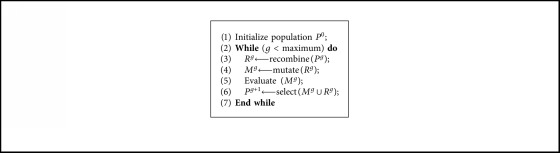
Pseudocode of GA.

**Algorithm 2:**
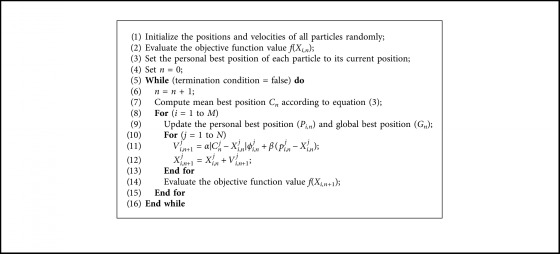
Pseudocode of RDPSO.

**Algorithm 3:**
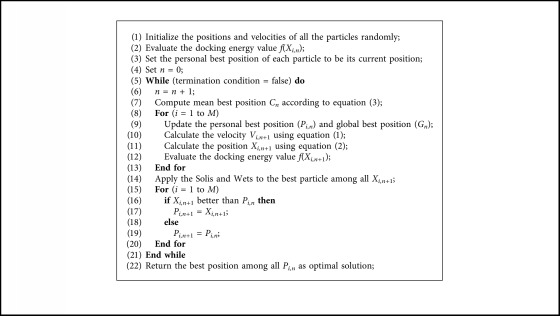
Pseudocode of LRDPSO.

**Table 1:** Comparison of the docking energy of complexes from LRDPSO and LGA.

Complex	Binding free energy (kcal/mol)	Vdw + Hbond + desolv energy (kcal/mol)	Electrostatic energy (kcal/mol)	Intermolecular energy (kcal/mol)	Internal energy (kcal/mol)
LRDPSO docking	−16.80	−16.86	−5.61	−22.47	−2.62
LGA docking	−12.74	−14.51	−3.90	−18.40	−4.33

Vdw = van der Waals; Hbond = hydrogen bonds; desolv = desolvation.

**Table 2:** Occupancy time of hydrogen bonds in the binding domain of the LRDPSO-docked complex at different temperatures.

Donor	Acceptor	Occupancy time (%) (300 K)	Occupancy time (%) (400 K)	Occupancy time (%) (500 K)	Occupancy time (%) (600 K)
ARG413-side	LYS3-side	100	100	100	100
LYS1-main	ASP419-side	100	100	92.79	92.39
ARG404-side	TYR2-side	100	72.53	2.92	1.51
ARG413-side	LYS3-main	100	54.61	76.1	68.13
LYS1-main	TRP416-side	100	100	31.5	13.51
CYS417-main	LYS1-main	89.58	86	77.86	66.16
LYS3-main	GLY415-main	89.35	83.7	65.39	46.06
LYS1-side	ASN506-side	87.25	37.96	3.19	2.46
LYS1-main	CYS417-main	85.43	76.36	39.27	49.8
TYR2-main	GLU32-main	79.58	50.61	45.56	20.99
LYS1-side	ASP419-side	66.58	61.30	64.63	35.96
VAL34-main	TYR2-main	63.90	68.97	34.23	30.07
LYS1-side	LEU504-main	59.15	26.52	2.95	3.95
LYS3-side	HSD371-side	52.33	0.61	0.16	1.04

**Table 3:** Occupancy time of hydrogen bonds in the binding domain of the LGA-docked complex at different temperatures.

Donor	Acceptor	Occupancy time (%) (300 K)	Occupancy time (%) (400 K)	Occupancy time (%) (500 K)	Occupancy time (%) (600 K)
ARG413-side	LYS3-side	100	100	100	85.78
ARG404-side	TYR2-side	100	100	11.58	5.8
LYS1-main	TRP416-side	100	100	67.93	14.29
ARG413-side	LYS3-main	100	100	93.55	51.71
LYS1-main	ASP419-side	94.37	100	99.3	46.38
CYS417-main	LYS1-main	92.25	87.1	80.22	41.29
LYS1-main	CYS417-main	85.62	67.95	61.67	24.05
LYS1-side	ASP419-side	83.87	86.03	62.23	34.33
LYS3-main	GLY415-main	80.48	62.68	61.62	30.8
VAL34-main	TYR2-main	74.75	80.47	20.37	27.76
TYR485-side	LYS3-side	70.33	11.2	16.12	3.4
TYR2-main	GLU32-main	68.58	44.52	58.4	21.9
LYS1-side	HSD161-side	61.48	8.3	34.8	1.14

**Table 4:** The average number of hydrogen bonds in both complexes in different temperature simulations.

Complex	Average number (300 K)	Average number (400 K)	Average number (500 K)	Average number (600 K)
LRDPSO-docked complex	208	200	182	155
LGA-docked complex	216	198	185	156

## Data Availability

The data used to support the findings of this study are included within the article.
